# Uncovering the True Diagnosis: A Case Report of Multilocular Peritoneal Inclusion Cyst

**DOI:** 10.7759/cureus.81724

**Published:** 2025-04-04

**Authors:** Maya Al-Saghir, Korina Gaishauser, Zaid R Al-Wahab

**Affiliations:** 1 Obstetrics and Gynecology, Corewell Health William Beaumont University Hospital, Royal Oak, USA

**Keywords:** cystic mesothelioma, lymphangioma, mpic, multicystic peritoneal mesothelioma, multilocular peritoneal inclusion cyst, peritoneal inclusion cyst, pic

## Abstract

Peritoneal inclusion cysts (PICs) are rare, benign cystic tumors predominantly found in premenopausal females. They often present diagnostic challenges due to their asymptomatic nature and non-specific imaging features, requiring biopsy and immunohistochemistry for a definitive diagnosis. This report presents a case of a large, multilocular PIC initially misdiagnosed as a lymphangioma.

A 29-year-old asymptomatic nulliparous female presented for follow-up after an abdominal ultrasound, conducted during a complicated cystitis workup, incidentally revealed a notable fluid collection adjacent to the spleen. Computed tomography of the abdomen and pelvis showed a complex cystic lesion in the left upper quadrant, measuring 7.6 x 11.7 cm. The finding was interpreted as a lymphangioma. Over six months, the lesion doubled in size, prompting further evaluation and doxycycline sclerotherapy. Despite eight months of treatment, the patient began to experience persistent symptoms of abdominal pressure, sharp groin pain, and decreased appetite. Magnetic resonance imaging revealed that the lesion had grown to 7.0 x 17.1 x 34.6 cm. An incisional biopsy confirmed PICs through pathology and immunohistochemistry. Genetic testing for hereditary cancer was negative. The patient underwent extensive surgical resection involving multiple organs, ultimately achieving no gross residual disease.

This case underscores the diagnostic challenges posed by PICs and the necessity of biopsy for accurate diagnosis, differentiating them from lymphangiomas. It emphasizes the importance of a multidisciplinary approach and individualized treatment plans when managing PICs. Continued research and long-term follow-up are essential for refining treatment strategies for this rare condition.

## Introduction

Peritoneal inclusion cysts (PICs) are rare, benign cystic tumors located in the pelvic region that arise as a result of non-neoplastic reactive mesothelial proliferation [1]. They are predominantly found in premenopausal females with hormonally active ovaries [1]. The diagnosis of PICs can be challenging due to their asymptomatic nature and non-specific features on diagnostic imaging. A definitive diagnosis requires a biopsy, followed by subsequent immunohistochemistry [2].

We present a case of a premenopausal female with a large multilocular PIC, initially misdiagnosed as a lymphangioma. She had no history of pelvic surgery, pelvic trauma, endometriosis, or pelvic inflammatory disease.

## Case presentation

A 29-year-old nulliparous female presented to our Emergency Department with flank pain, fever, and headache. She had recently been diagnosed with complicated cystitis and treated with antibiotics. Her medical history included melanoma, an abnormal Pap smear of the cervix, herpes zoster, and anxiety. Her surgical history was limited to the local resection of melanoma on her upper chest.

**Figure 1:**
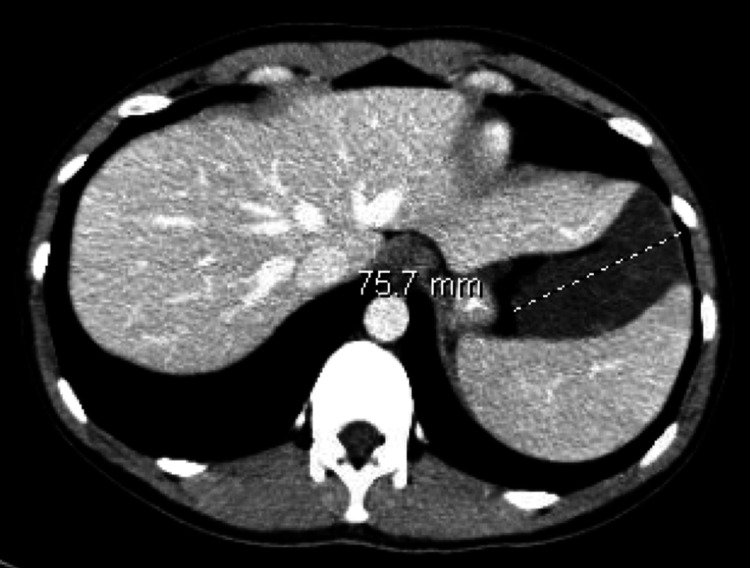
CT Abdomen/Pelvis With Intravenous (IV) and Oral Contrast Left-sided subdiaphragmatic cystic lesion measuring 7.6 cm in transverse dimension is noted below the diaphragm between the liver and the spleen. CT, Computed tomography

Abdominal ultrasound revealed a notable fluid collection with septations adjacent to the spleen. A follow-up computed tomography (CT) of the abdomen and pelvis showed a complex cystic lesion in the left upper quadrant, measuring 7.6 x 11.7 cm (Figure 1). The CT was reported as a lymphangioma. The patient had no associated symptoms at this time, and her physical exam was unremarkable. No therapeutic intervention was initiated at that time, and she was scheduled for a follow-up CT scan in six months.

At her appointment six months later, the patient noted feeling pressure and a pulling sensation on the left side of her abdomen, which she rated as 4/10 for pain. A repeat CT scan showed that the cystic lesion had almost doubled in size, now measuring 11 x 22 cm, with extension into the hepatorenal space. Due to the rapid increase in the size of the lesion, aspiration and magnetic resonance imaging (MRI) were performed. Ultrasound-guided aspiration revealed 33 mL of clear yellow fluid, which was sent to pathology and revealed no evidence of malignant cells. The MRI demonstrated similar findings to the previous CT scan, with a large T2 hyperintense cystic mass extending from the spleen on the left to the left hemipelvis, along with enhancing septations. The differential diagnosis at this time continued to favor a lymphangioma.

Due to concerns about difficulty with resection, including the potential for significant organ damage and recurrence, it was decided that she receive doxycycline sclerotherapy over a span of eight months. Post-sclerotherapy, ultrasound revealed a decrease in the size of the inferior aspect of the cystic lesion; however, the size remained unchanged in the superior aspect. The patient had worsening symptoms of abdominal pressure, sharp groin pain radiating to the abdomen, discomfort with bowel movements and urination, and decreased appetite. An MRI was conducted to reevaluate the cyst, and the results revealed that the lesion was continuing to grow in size, now measuring 7.0 x 17.1 x 34.6 cm. The MRI also noted mass effect and rightward displacement of the stomach, colon, small bowel, and uterus.

In light of the patient’s unsuccessful response to sclerotherapy and worsening symptoms, she was referred for hereditary cancer risk evaluation, and a decision was made to perform a tissue biopsy. Genetic testing, which analyzed 77 genes associated with hereditary cancer, yielded negative results, with no pathogenic mutations identified. The biopsy, obtained through a 4 cm low midline incision, revealed multilocular PICs, leading to a revision of the prior clinical and radiographic diagnosis. Cytology remained negative for malignancy, and immunohistochemistry results further supported the diagnosis of PICs.

Surgical planning thus began for the removal of the PICs. An interdisciplinary team, including Gynecology Oncology and General Surgery, was formed in order to achieve maximal debulking of the mass while preserving the patient's fertility. The goal of this surgery was not only to alleviate the patient's current symptoms, but also to enhance the prospects of successful future pregnancies.

**Figure 2:**
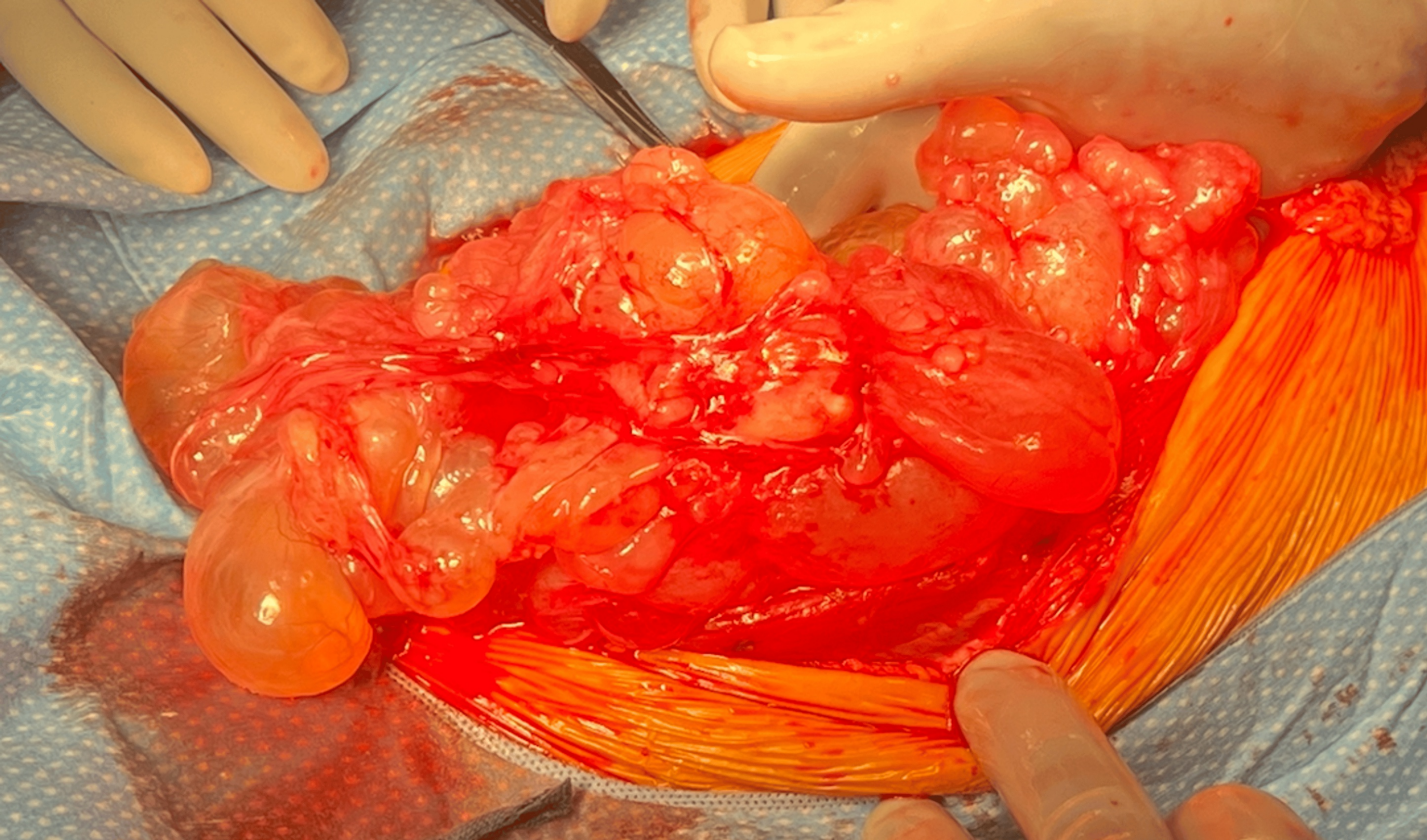
Intraoperative View of Cystic Lesions Image taken intraoperatively during the removal of cystic lesions.

**Figure 3:**
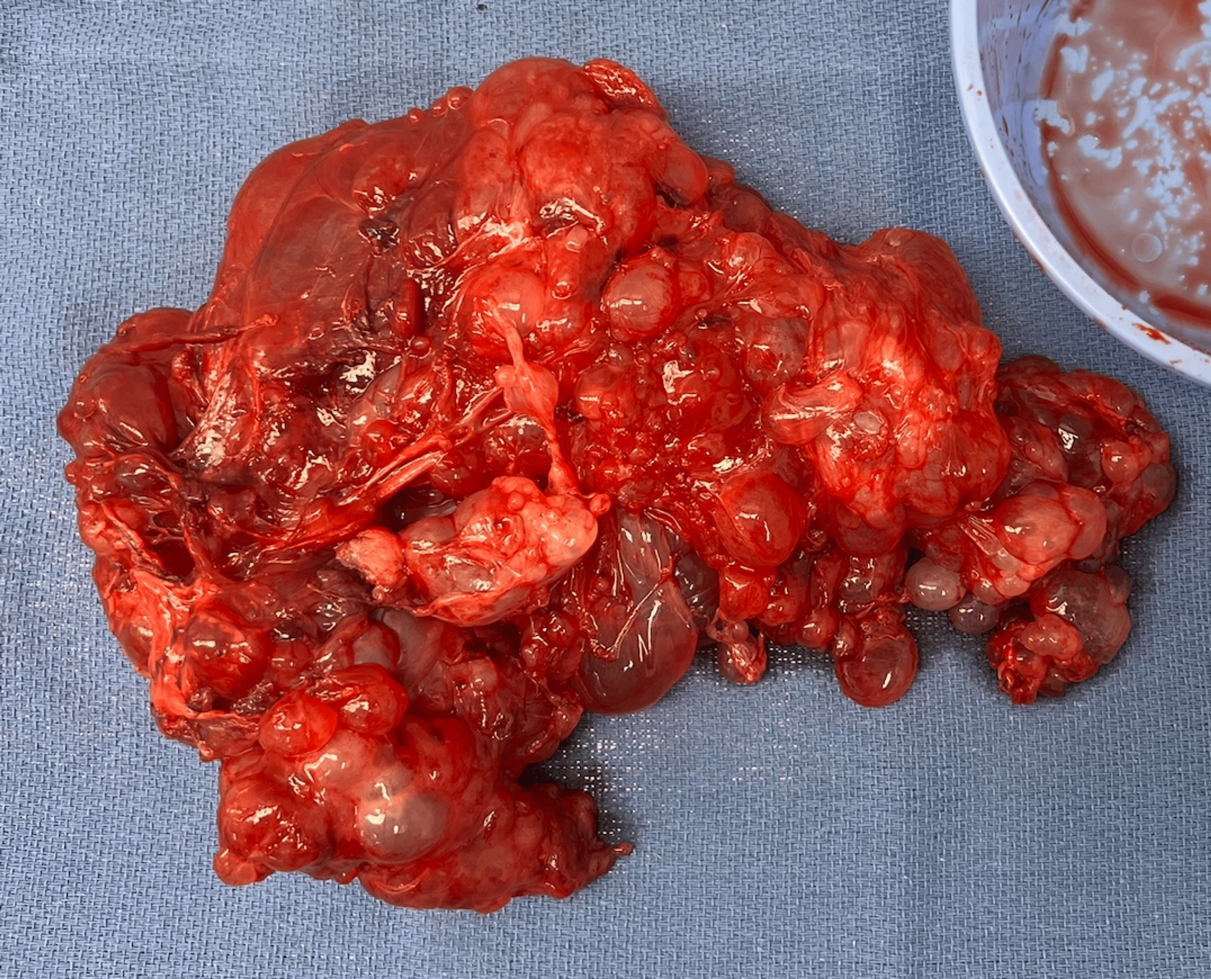
Gross Specimen of Cystic Structure With Attached Omentum Multiloculated cystic structure with an attached portion of omentum, weighing 941 g and measuring 28.0 x 13.5 x 8.5 cm.

**Figure 4:**
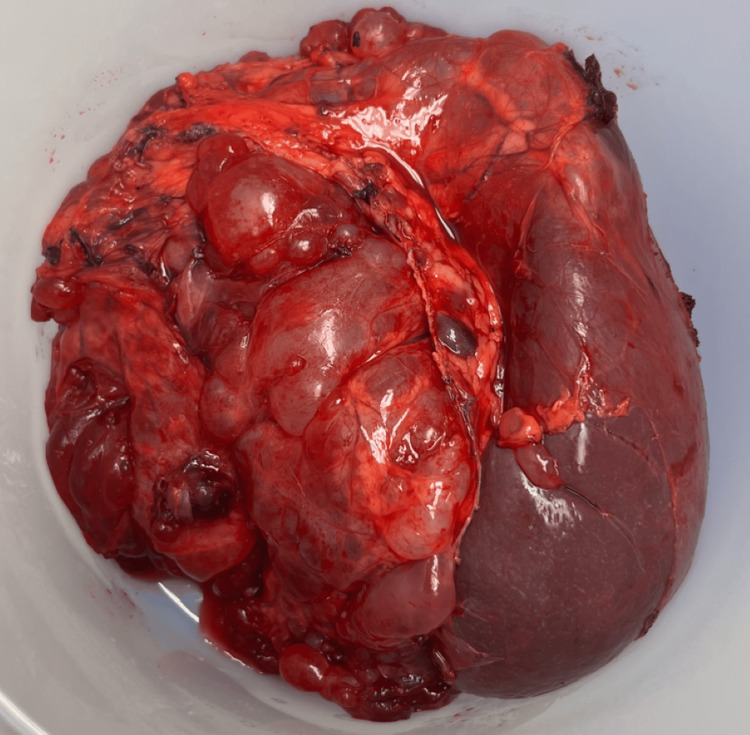
Gross Specimen of Mass Adherent to Spleen Multicystic mass densely attached to the spleen.

Ultimately, the patient underwent an exploratory laparotomy, excision of pelvic peritoneal cysts, resection of the falciform ligament, peritonectomy, omentectomy, splenectomy, appendectomy, two hepatic wedge resections (segment 2 and segment 7), and mobilization of the splenic flexure. Intraoperative findings showed multiple cystic lesions densely adherent to the spleen, small bowel, large bowel, liver, omentum, ovaries, and anterior abdominal wall (Figures 2-4). At the end of the procedure, there was no evidence of any gross residual disease in the abdomen and pelvis, and no evidence of any injuries to any organs in the abdomen and pelvis. Two weeks postoperatively, she was admitted for complications related to a small bowel obstruction, which resolved with nasogastric decompression and a Gastrografin challenge. Her recovery was otherwise uncomplicated, and she reported complete symptomatic relief.

## Discussion

PICs, alternatively referred to as multicystic peritoneal mesothelioma, are a rare benign cystic pelvic lesion, most often diagnosed in premenopausal females [2]. The predominant hypothesis suggests that these lesions arise as a peritoneal response secondary to inflammation or injury to the pelvis [2]. Pelvic surgery, endometriosis, and pelvic inflammatory disease are currently identified as significant risk factors, although they were absent in our patient’s case [1].

PICs are often asymptomatic or present with nonspecific symptoms such as chronic or intermittent lower abdominal pain, abdominal distension, tenderness, and changes in bowel movements or urination [1]. The lesion is often initially visualized using ultrasonography and typically appears as a multiseptate, anechoic cystic structure [1]. CT imaging can help evaluate the extent of the lesion, which characteristically presents with fluid attenuation and septa that enhance after intravenous (IV) contrast [1]. The presence of enhancing solid components within the lesion, peritoneal deposits, ascites, or calcifications is not characteristic of these lesions and should raise concern for malignancy [3]. MRI is most helpful in evaluating the relationship of the lesion to adjacent pelvic structures, with the lesion appearing hyperintense on T2-weighted images [2].

As seen in our case, radiologic evaluation has significant limitations in distinguishing PICs from other peritoneal masses, particularly lymphangiomas. Cystic lymphangiomas are congenital, benign, proliferative lymphatic malformations that can exhibit imaging features identical to PICs [4]. However, there are important clues that can aid in narrowing the differential diagnosis. Intraperitoneal cystic lymphangiomas are predominantly situated in the small bowel mesentery, whereas PICs are more commonly found in close proximity to the uterus, ovaries, and rectum [4]. Additionally, lymphangiomas are more prevalent in pediatric patients, with a higher incidence among males than females. The fluid contents of lymphangiomas can be either serous or hemorrhagic and are more likely to be chylous than those of PICs [4].

While imaging modalities provide valuable insights, particularly for surgical planning, the definitive diagnosis of PICs ultimately requires an incisional or excisional biopsy, as demonstrated in our case. A definitive diagnosis is achieved through biopsy and subsequent immunohistochemistry [2]. Positive mesothelial markers, such as cytokeratin 5/6, calretinin, and transcription factor WT, along with negative endothelial markers, such as CD31, CD34, and factor VIII, characterize PICs [2,4]. Conversely, lymphangiomas exhibit the opposite profile, showing negative mesothelial markers and positive endothelial markers. This distinction underscores the significance of biopsy in our patient’s definitive diagnosis [2].

The treatment for PICs is highly variable and, depending on the patient, may involve observation, drug therapies, drainage, sclerotherapy, surgical resection, or a combination of these approaches [2,3,5]. Although surgical resection has historically been the favored approach for PIC management, more recently, there has been a growing preference for conservative approaches [5]. This shift is, in part, due to the recurrence rate of PICs following surgical resection, reportedly as high as 50% [3,2]. Potential drug therapies include hormonal treatments, such as oral contraceptives, gonadotropin-releasing hormone agonists, and anti-estrogenic agents like tamoxifen [3,6]. The use of these therapies offers a promising avenue for those who may not be ideal candidates for surgery or when other interventions prove insufficient. Image-guided drainage, followed by sclerotherapy, is another treatment option that has worked for patients [3,7]. Despite advancing insights, a comprehensive understanding of PIC is still limited, necessitating ongoing research efforts to establish a more standardized treatment approach.

## Conclusions

We present a case of a large PIC in a premenopausal female, initially misdiagnosed as a lymphangioma. This case highlights the intricate diagnostic challenges and diverse treatment considerations associated with PICs. While imaging techniques provide valuable insights, they may not always be sufficient in differentiating between PICs and lymphangiomas. A biopsy is a crucial diagnostic tool and can serve as the differentiating factor for an accurate diagnosis. A multidisciplinary approach is necessary to develop an individualized treatment plan that addresses all of the patient’s unique care goals. Continued research is imperative, and longitudinal follow-up is essential to refine treatment algorithms for this rare condition.
